# Rapid, cost-effective, sensitive and quantitative detection of *Acinetobacter baumannii* from pneumonia patients

**Published:** 2011-12

**Authors:** B Nomanpour, A Ghodousi, A Babaei, HR Abtahi, M Tabrizi, MM Feizabadi

**Affiliations:** 1 Department of Microbiology, School of Medicine, Tehran University of Medical Sciences, Tehran, Iran; 2 Department of Anesthesiology, Shaheed Rajaei Cardiovascular, Medical & Research Center, Vali- Asr Avenue Tehran- Iran; 3 Department of Internal Medicine, School of Medicine, Tehran University of Medical Sciences, Tehran, Iran; 4 Department of Medical Genetics, School of Medicine, Tehran University of Medical Sciences, Tehran, Iran; 5 Pediatric Infection Diseas Research Center, Tehran, University of Medical Science, Tehran, Iran

**Keywords:** HAP, VAP, *Acinetobacter baumannii*, Real time PCR, Pneumonia

## Abstract

**Background and Objectives:**

Pneumonia with *Acinetobacter baumannii* has a major therapeutic problem in health care settings. Decision to initiate correct antibiotic therapy requires rapid identification and quantification of organism. The aim of this study was to develop a rapid and sensitive method for direct detection of *A. baumannii* from respiratory specimens.

**Materials and Methods:**

A Taqman real time PCR based on the sequence of *bla*_*oxa-51*_ was designed and used for direct detection of *A. baumannii* from 361 respiratory specimens of patients with pneumonia. All specimens were checked by conventional bacteriology in parallel.

**Results:**

The new real time PCR could detect less than 200 cfu per ml of bacteria in specimens. There was agreement between the results of real time PCR and culture (Kappa value 1.0, p value<0.001). The sensitivity, specificity and predictive values of real time PCR were 100%. The prevalence of *A. baumannii* in pneumonia patients was 10.53 % (n=38). Poly-microbial infections were detected in 65.71% of specimens.

**Conclusion:**

*Acinetobacter baumannii* is the third causative agent in nosocomial pneumonia after *Pseudomonas aeroginosa* (16%) and *Staphylococcus aureus* (13%) at Tehran hospitals. We recommend that 104 CFU be the threshold for definition of infection with *A. baumannii* using real time PCR.

## INTRODUCTION

Strains of *Acinetobacter baumannii* have emerged as causative of nosocomial infections, posing high morbidity and mortality at hospitals throughout the world (1). The organism has also recently been isolated from cases with community acquired pneumonia (CAP) (2). Although the virulence factors of *Acinetobacter* have not been fully determined, (2–4), it has been identified as one of the three greatest threats to admitted patients at hospitals (1). The organism is able to rapidly develop resistance to all recommended antibiotics far exceeding *P. aeroginosa* capabilities (5).

Successful treatment of nosocomial pneumonia resulting from this “*very successful pathogen*” (3) requires detection of *A. baumannii* in clinical specimens (1, 6, 7). The guidelines of the American Thorax Society/ Infection Disease Societies of America (ATS/IDSA) recommend empirical therapy for pneumonia at admission only, and the microbiologic detection of pathogens should be initiated as soon as possible. Besides accurate detection, quantification of *A. baumannii* particularly in pneumonic samples for effective, timely, and accurate therapy is critical (8). This is the reason why the guidelines for hospital associated pneumonia (HAP) and ventilator associated pneumonia (VAP) patients try to prevent inappropriate and overuse of prescription antibiotics. In presence of pneumonia symptoms (body temperature >38°C, leukocytosis or leukopenia and purulent exudate) and detection of causative agent, empirical therapy can be initiated (ATS/IDSA guideline) (8, 9). Differentiation of colonization from true pneumonia in hospitalized patients is difficult. However, isolation of 104cfu/ml from clinical respiratory specimens has been defined as a cut-off for infection (2). Despite this definition, literature is scarce and there is controversy concerning the method for determination of *A. baumannii* threshold when the physicians perform pathogen-directed therapy. Conventional bacteriology for detection and quantification of *A. baumannnii* is time consuming and its sensitivity is lower than molecular techniques such as real-time PCR; therefore, real time PCR can also be regarded as more cost-effective. Data concerning rapid diagnosis of *A. baumannii* directly from clinical specimens in particular respiratory specimens is rare. Real-time PCR can overcome this problem since it quantitatively detects *A. baumannii* from pneumonic patients (10, 11). The technique takes less than two hours and produces results with high sensitivity and specificity. We have previously found that different clones of *A. baumannii* with multi-drug resistant (MDR) and pan-drug resistant (PDR) phenotypes exist at Tehran Hospitals (12). In this study, we designed a TaqMan-based real-time PCR assay for rapid detection and quantification of *A. baumannii* from the respiratory specimens of patients with pneumonia and compared the results with those obtained by conventional bacteriology. To our knowledge, this is the first study conducted in the Middle East in which a molecular technique was used to differentiate colonization from infection by introducing a cut-off that detects infections by *A. baumannii* in hospitalized patients.

## MATERIALS AND METHODS

**Clinical specimens and isolates.** A total of 361 clinical specimens including bronco alveolar lavage (BAL), sputum, tracheal aspirates and throat swabs were collected from 2 teaching and 2 private hospitals of Tehran, Iran (Table 1). The BAL and tracheal aspirate specimens (218 of BAL and 86 tracheal aspirates) were collected from patients at ICU or RCU, of which 80 were collected from patients with ventilated associated pneumonia (VAP). Sputum and throat swab specimens occupied 57 total specimens, of which 45 were taken at emergency units during January 2009- February 2010. Hospital associated pneumonia was diagnosed based on the ATS/ IDSA guideline (9) i.e., pneumonia was detected by infiltration on chest x-ray, clinical findings (body temperature more than 38.5 or less than 36°C and leukocytosis or leukopenia) and patients who developed a new pneumonia infection on mechanical ventilation for more than 96 hours, diagnosed as having VAP (13). Information concerning the age, symptoms and underlying diseases of patients with pneumonias in this study is shown in Table 2.

**Table 1:** Number of Clinical specimens and isolates.

Specimens	BAL	Tracheal aspirate	Sputum & swab
HAP	201	21	12
VAP	17	63	0
CAP	0	2	45
Total	218	86	57

**Table 2:** Pneumonia Patient Characteristics.

Characteristic	HAp (%)	VAp (%)	CAp (%)
Age (Mean)	35−78 (56.4±2.3)	14−68 (58.3±2.9)	8−59 (39±1.1)
Body temperature ≥ 38.5°C	234	79	47
Leukocytosis	234	79	47
Background disease	–	–	–
Cardiac surgery	8 (3.42)	29 (36.25)	–
Heart failure	48 (20.51)	23 (28.75)	5 (10.64)
Transplanted (most kidney)	5 (2.14)	4 (5)	–
Diabetic	96 (41.03)	16 (20)	29 (61.7)
COPD	24 (10.25)	5 (6.25)	7 (14.89)
Cancer, Surgery and other	53 (22.65)	3 (3.75)	6 (12.77)
Total	234	80	47

*Patients who had more than one of these back ground disease, was grouped in one category.

From every BAL or sputum specimens, 2 ml was kept at −20°C for real time PCR and Swab of patient was suspended in a tube containing two milliliter of 0.85% NaCl, and the remainder was used for bacterial culture and identification using standard methods as described previously. In brief, a calibrated loop (0.01µl) was used to streak the specimens on the plates containing blood agar and the colonies were counted after overnight incubation (14). Identification of isolates as *A. baumannii* was based on Gram staining, colonial morphology and biochemical properties (14). Culture and counting of the resulted colonies were done in duplicate. Count reporting for culture based on Colony Forming Unit (CFUs) were 1,000–10,000–50,000 and 100,000 (14, 15).

**DNA extraction:** DNA was extracted using enzyme digestion and the phenol chloroform method as described previously (16). Five microliter aliquots of extracted DNA solution were used as template in Real Time PCR assay (16).

**Standards, sensitivity and specificity:***A. baumannii* (NCTC 19606) and human DNA were used as positive and negative controls respectively. From the overnight growths of *A. baumannii*, serial dilutions containing 102 CFU/ml to 108 CFU /ml were prepared in 0.85% NaCl. They were used for drawing standard curve and to determine the sensitivity of real time PCR by comparing Standard tube to turbidity of 0.5 McFarland (approximate count 1.5×108 CFU/ml bacteria) and using spectrophotometer (Eppendorf, Hamburg, Germany) as described previously (16). When culture-based colony count and spectrophotometry differences were<1%, it was considered as standard tube (It had calculated colony forming unit/ ml). DNA was extracted from 200µl of the standard tubes and specimens (16).

Bacterial DNA from *Pseudomonas aeruginosa* (ATCC 49189) *Stenotrophomonas maltophilia* (NCTC10257), *Klebsiella pneumoniae* (NCTC 5056), *Escherichia coli* (NCTC 21157), *Staphylococcus aureus* (ATCC 29213), *Streptococcus pneumoniae* (NCTC11910), *Legionella pneumophila* (NCTC 11192), *Mycoplasma pneumoniae*, (NCTC 10119) and human DNA were used as negative controls in real time PCR. Beta actin gene (primer and probe) was used as internal control with every sample to detect PCR inhibitor as recommended (17). The human white blood cell DNA was used as negative control in this study since our primers and probe do not react with human genome.

The specificity of the developed Real Time PCR was assessed using the above standard bacterial strains as well as the specimens that were positive for other microorganisms. For plotting standard curve, Real Time PCR assays were performed in duplicate.

**Primer design.***bla*_*oxa*-51_ gene sequence which is specific to *A. baumannii*, was selected based on our previous study (12). Conserved sites were identified by multiple alignments in NCBI and a primer pair and probe were designed using primer 3 plus (http://www.bioinformatics.nl/cgi-bin/primer3plus/primer3plus.cgi). The specificity of the candidate primers and probe for all bacterial sequences in the database were verified by FASTA analysis. (http://www.ncbi.nlm.nih.gov/GenBank)

For amplification of the *bla*_*OXA*-51_, forward primer: Oxa51-F: 5-CTCGTGCTTCGACCGAGTAT-3, reverse primer: Oxa51-R: 5-AACCAACACGCTTCACTTCC-3 (20bp) and probe: Oxa_ probe: 5- Texas Red –ATGAAAGCTTCCGCTATTCCGGTT-BHQ3-3 (24bp, Tm=66) were designed. The size of amplicon was 247bp.

**Real-time PCR.** Amplification and detection were performed in LinGene K Real Time PCR apparatus (Bioer, Hangzhou, P.R. China). The reactions consisted of 0.2 µM of each primer and probe, 0.2 µM dNTP, 1.5µM MgCl^2^, 1.2U of DNA polymerase (mi Taq- Metabion, Martinsried, Germany), 5µ of 10x PCR buffer, and 5 µl of the template DNA in total volume of 45µl with double distilled water. The cycling program was 95°C for 10 min and then 35 cycles of 10s at 95°C (denaturation) followed by 45s at 60°C with fluorescent collection (annealing and extension). Every sample was run in duplicate and the mean was reported. The efficacy of PCR (E) was calculated using the slope of standard curve in the equation: E=10 (-1/ slope) -1 as described by Pfaffl (18). Comparisons of culture and real time PCR were performed by using Cohen′s Kappa test. The SPSS 13.0 software program was used for statistical analysis of the data.

## RESULTS

**Culture and characterization of isolates.** Of 361 clinical specimens, 38 yielded *Acinetobacter* in culture. The Gram-negative non-motile coccobacilli that grew at 37 and 44°C were identified as *A. baummanii* if they were negative in oxidase and DNase tests but positive for decarboxylation of lysine, production of Xylosidase (by ONPX disk) and acidification of glucose and malate in OF media. The identity of all 38 isolates were re-confirmed by PCR based on the presence of *bla*_ox-51_ as this gene previously was proved to be specific for identification of Iranian strains of *A. baumanni* (12). The results of bacteriology for 274 specimens were also positive for *P. aeroginosa* (n=58), *E. coli* (n=20), *K. pneumonia* (n=26) and others. *Staphylococcus* spp. (n=67) and other Gram positive cocci (n=65) and the yeasts (n=17) grew from the clinical specimens too (Table 3). Poly-microbial growth was observed in 235 specimens (65.71%) including those yielding more than 2 different bacteria and yeast in culture. Counting of *A. baumannii* colonies demonstrated the existence of less than 500 to more than 105 CFU/ml in positive cultures (Table 3).

**Table 3:** Results of culture and real-time PCR for *Acinetobacter baumannii* detected from patients with pneumonia at Tehran Hospitals.

patients ID	CFU in Culture	Copy numbers inRT-pCR^1^	poly microbial infection
L1	10,000	7.36E+03	*P. aeroginosa*
L46	100,000	2.40E+5	*S. aureus*
L51	100,000	2.41E+05	
L59	50,000	9.62E+04	*E. fecalis*
L76	100,000	4.98E+07	*P. aeroginosa*
L99	50,000	6.70E+04	
R9	50,000	2.37E+04	*E. coli K. pneumoniae*
R19	100,000	2.61E+07	*E. fecalis S. epidermidis*
R22	100,000	1.75E+06	
R25	50,000	7.20E+05	*P. aeroginosa E. fecalis*
R27	100,000	2.90E+05	
R34	50,000	6.34E+04	*P. aeroginosa*
R39	10,000	6.73E+03	
R45	100,000	3.21E+05	
E20	10,000	7.41E+05	
E30	50,000	2.81E+06	*S. epideremidis*
E39	50,000	2.17E+04	*S. aureus*
E43	10,000	1.24E+04	
E48	10,000	3.42E+04	*S. maltophilia P. aeroginosa*
E65	100,000	8.07E+09	*A. niger*
E67	100,000	7.25E+09	
E70	5,000	8.97E+03	
E73	100,000	8.46E+08	*Candida spp. S. epidermidis*
E74	10,000	2.31E+04	
E77	5,000	3.54E+03	*S. aureus*
E87	50,000	8.21E+04	
E88	50,000	4.67E+04	
E89	100,000	9.02E+10	*S. epidermidis*
E92	10,000	1.57E+04	*S. aureus*
E95*	< 500*	756	*S. aureus*
E97	100,000	5.98E+08	*Alcaligenes sp*.
M3	< 5,000	3974	
M11	100,000	4.29E+07	
M27	10,000	5. 15E+04	
M44	50,000	4.26E+06	*K. pneumoniae*
M 68	100,000	1.46E+09	
M82	100,000	1.38E+07	*S. maltophila*
CAP	10,000	7750	

1 1-Real time-PCR was run in duplicate.

* Antibiotic regimen was changed to polymyxin B before sampling.

Five VAP patients infected with *A. baumannii* expired. In one of them the isolated *A. baumannii* showed PDR phenotype. Similarly, a lung transplanted patient who had polymicrobial infection was also infected with a PDR strain. However, in this patient as well as 6 other expired VAP cases *Staphylococcus aureus* was the main cause of death. Overall, 19 cases with VAP (23.75%) were expired. On the other hand, *A. baumannii* was the main cause of deaths (n=4) in patients with HAP. Of 234 patients with HAP, 22 (9.40 %) were expired.

**Clinical finding.** Infiltration of lung (chest X-ray) was included as a criterion for hospital admission. All patients infected with *A. baumannii* had temperature body more than 38.5°C and leukocytosis. A lung transplanted patient had body temperature lower than 36°C and leukopenia (less than 4000). Gram staining of direct smear in specimens showed more than 12-14 White blood cells (WBC) per oil-high power field.

**Culture quantification and Real time PCR.** All specimens were cultured on Muller Hinton agar (10cm diameter plate) and semi-quantified. Real time PCR detected 38 samples as being positive for *A. baumannii* in culture and counted 790 and 3936 cfu/ml in 2 specimens from HAP patients which showed less than 104cfu/ml in culture. This technique counted less CFUs than culture from CAP specimens (Table 3).

Standard curve of duplicated every dilution had slope -3.39±0.1 and R2>0.99 with SD 0.1. Efficacy of PCR test was 97.25 % (E=10 (-1/slope)-1) (18). Results of real time PCR and quantification of every sample are shown in Table 3. The sensitivity, specificity, and predictive values were 100%. Amplification of target genes from *A. baumannii* in serial dilutions ranged from 109 CFU /ml to 102 CFU /ml and showed that the technique can detect every calculated tube count with SD<1%. The results were performed in duplicate. There was agreement between the results of culture and real-time PCR such that Cohen′s Kappa value was 1.0 (SD 0.00, p<0.001).

## DISCUSSION

*Acinetobacter baumannii* is the second most commonly isolated non-fermenting bacteria in healthcare associated infection and third infective organism at ICUs causing mortality rates of 26-68% (1, 19). This organism which has been nominated as the *“bad bug”* (20, 21), possesses intrinsic and acquired mechanisms of resistance to various classes of antibiotics (5). The multi-drug resistant (MDR) and pan-drug resistant (PDR) *A. baumannii* has also been described as a *“very successful”* pathogen for escaping from every human challenge (6, 7, 20). Rapid detection of *A. baumannii* from patients with HAP or VAP and severe pneumonia is critical because *A. baumannii* may rapidly become resistant and life threatening (1). To accurately diagnose pneumonia and consequently prevent misuse or overuse of antibiotics (7), microbiological methods including quantitative culture and susceptibility pattern of isolated organisms should be implemented in diagnosis and therapeutic policy (2, 22). Most efforts for detection and identification of *A. baumannii* are based on phenotypic characteristics of isolated organisms and this takes at least three days to yield results. On the other hand, early detection and adequate treatment within 6–36 hours is critical for ICU patients (23, 24, 25). Conventional PCR is not as sensitive and specific as real-time PCR because of carry-over contamination and false positive and false negative results due to the nature of post common PCR detection methods. In addition, it also takes more time.

Real-time PCR is rapid and sensitive. It gives results with high specificity but not at expense of sensitivity and so it can be used routinely in many laboratories. Despite the importance of accumulating data about *A. baumannii*, rapid detection method of this bacterium was scarce (3). So, we designed a TaqMan-based real-time PCR assay for detection and quantification of *A. baumannnii* from clinical specimens using the *bla*oxa51 gene. This gene was first reported by Brown *et al*. (26) and later was found to be specific for *A. baumannii* (3, 12, 27). Knowing this background, we designed primers and TaqMan probe with 100% specificity for direct detection and quantification of *A. baumannii* from respiratory specimens. This new rapid technique was used in challenge with a variety of Gram positive and Gram negative bacteria that may be present in respiratory specimens. Sensitivity of primer probe was checked in serially diluted standard tubes. It could detect 200 cfu/ml of bacteria in samples.

Early appropriate antimicrobial therapy decreases mortality rates. It requires quantitative culture with threshold of 104 cfu/ml of BAL or 103 cfu/ml for protected brush specimens to differentiate infection from non-infectious but suspected patients according to ATS/IDSA guidelines (28). For specimens other than BAL (tracheal aspirate, sputum and throat swab), the quantification should be compared with clinical findings and chest infiltrates. In presence of symptoms, it is suggested that 104cfu/ml of bacteria is compatible with infection although sputum and tracheal aspiratyield more than 10^5^cfu/ml bacteria in infection cases. Detection of *A. baumannii* at low numbers may be indicative of infection and antibiotic therapy can possibly be a reason in such cases. For example, two patients (E95 and M3 isolates) had lower than 104cfu/ml *A. baumannii* in their specimens (They were counted by real-time PCR, 795 and 3937 cfu/ml respectively). M3 was isolated from a transplant patient who was under antibiotic therapy and its specimen was quantified for monitoring and efficacy of therapy. The antibiotic regimen of the second patient (E95) had been changed in 24 hours before sampling. Fabregas et al. (1999) suggested that counting of 103-105cfu/ml in specimens from lung autopsy is compatible with pneumonia (29). Torres and El-Ebiary reviewed 23 studies and reported threshold of colonization and infection of BAL specimens to be 104cfu/ml or lower (30). The results of our real-time PCR also suggest that the threshold for every sample from HAP and VAP should be considered as 104CFUs/ml. Based on the *Iceberg* model of *A. baumannii* colonization (3), and its potential for intrinsic and acquired resistance, we recommend appropriate therapy be initiated when real-time PCR detects even less than 103 cfu/ml. A recent survey on 55,330 isolates in the USA demonstrated that *A. baumannii* becomes carbapenem resistant with faster growth when it is colonized in the respiratory tract (31). This finding supports our recommendation to start antimicrobial therapy when the bug has colonized the lower respiratory tract at lower numbers than threshold. Obviously, mechanical intubation and potential of introducing bacteria to the lower respiratory tract and expression of silent intrinsic resistance genes under antibiotic pressure may help faster spreading of resistant isolates. The prevalence of *A. baumannii* at Tehran hospitals (10.2%) was similar to Korea (9%) (24), but lower than India, Taiwan, Bahrain and Turkey (p<0.001) (24) and polymicrobial infections were found in 13.3% of all specimens which is close to other studies (9). *A. baumannii* was found as the third bacterium after *P. aeruginosa* and *S. aureus* isolated from HAP and VAP cases, but a higher mortality in patients with VAP were associated with *S. aureus* and *A. baumannii* (7 and 6 cases, respectively). MDR and PDR isolates explosion of *A. baumannii* in medical care units is a global problem. To control this “*very successful pathogen*”, rapid detection is very important. Care should be taken in pneumonia cases with less than 104 cfu/ml of *A. baumannii*. Otherwise, it can impose great costs to the public health.
